# Integrating active surveillance and HPV vaccination for high-risk CIN2: a strategy to reduce disease progression

**DOI:** 10.3389/fmed.2026.1767329

**Published:** 2026-03-16

**Authors:** Kaiyang Geng, Siyu Bai, Ruizhen Liu, Longyu Jia, Yan Zhai, Zhihua Sun, Ruili Jiao, Xue Li, Jun Liu, Huimin Bai

**Affiliations:** 1 Department of Obstetrics and Gynecology, Beijing Chao-yang Hospital, Capital Medical University, Beijing, China; 2 Department of Obstetrics and Gynecology, Fuxing Hospital, Capital Medical University, Beijing, China; 3 Department of Obstetrics and Gynecology, Beijing Chaoyang District Maternal and Child Health Care Hospital, Beijing, China; 4 Department of Pathology, Beijing Chao-yang Hospital, Capital Medical University, Beijing, China

**Keywords:** active surveillance, cervical intraepithelial neoplasia grade 2, disease progression, HPV16, human papillomavirus vaccination, risk stratification

## Abstract

**Objective:**

This study aimed to identify risk factors associated with disease progression in women with cervical intraepithelial neoplasia grade 2 (CIN2) undergoing active surveillance and to evaluate the protective effect of human papillomavirus (HPV) vaccination administered after diagnosis.

**Methods:**

This single-center, retrospective cohort study included 510 women (mean age 30.15 ± 5.82 years, range 18–61) with histologically confirmed CIN2 who chose active surveillance between January 2012 and March 2021. Patient demographics, clinical characteristics, and HPV vaccination status were collected and analyzed. Primary and secondary outcomes were complete regression, persistence, and progression of disease (to CIN3, adenocarcinoma *in situ* [AIS], or invasive cancer). Univariate and multivariate logistic regression analyses, along with Cox proportional hazards models, were applied to assess risk factors.

**Results:**

Over a mean follow-up period of 37.0 months, 65.9% (336 of 510) of patients achieved complete regression, 10.8% (55 of 510) exhibited persistent CIN2, and 6.3% (32 of 510) experienced disease progression. A multivariate analysis identified HPV16 infection (OR 4.07, 95%CI 1.88–8.81) and having more than three pregnancies (OR 4.38, 95%CI 1.63–11.79) as independent risk factors for progression. Notably, HPV vaccination administered after CIN2 diagnosis was found to be an independent protective factor (OR 0.29, 95%CI 0.08–0.98). The Kaplan–Meier estimated 5-year progression rate was 72% (5 of 13 patients) in the high-risk subgroup (unvaccinated, HPV16-positive women with more than three pregnancies), compared with only 7.6% (27 of 497 patients) in the low-risk subgroup (vaccinated, HPV16-negative women with three or fewer pregnancies).

**Conclusion:**

Active surveillance is a safe and effective management strategy for the majority of women with CIN2. Risk stratification using HPV16 infection status and pregnancy history is crucial for personalized management. HPV vaccination after CIN2 diagnosis is strongly recommended, as it significantly reduces the risk of disease progression.

## Introduction

Cervical cancer (CC) remains a major public health concern for women globally, with an estimated 565,000 new cases and 280,000 deaths reported annually (1). Extensive research has established a strong causal relationship between persistent infection with high-risk human papillomaviruses (HR-HPVs) and the development of CC (2). The progression from HR-HPV infection to invasive carcinoma typically occurs over a period of 8–10 years, providing a critical window of opportunity for intervention through the management of precancerous lesions, collectively referred to as cervical intraepithelial neoplasia (CIN) (3).

Cervical intraepithelial neoplasia grade 2 (CIN2) presents a particular clinical challenge. Its pathological diagnosis shows poor inter-observer reproducibility, with concordance rates below 50%, underscoring the heterogeneous nature of these lesions (4). According to the 2014 World Health Organization (WHO) classification, a two-tiered system is recommended: low-grade squamous intraepithelial lesions (LSILs, encompassing CIN1) and high-grade squamous intraepithelial lesions (HSILs, combining CIN2 and CIN3). Given that HSILs carry a high risk of progression to CC, interventions such as cervical conization are often necessary (5). However, this procedure carries reproductive risks, including cervical insufficiency, miscarriage, preterm birth, and premature rupture of membranes in future pregnancies.

Moreover, evidence indicates that a substantial proportion of CIN2 lesions may undergo spontaneous regression (6), and the progression of CIN appears to be influenced by multiple factors that are not yet fully understood (7). Consequently, active surveillance has emerged as a more conservative management approach for selected CIN2 patients, aiming to balance effective cancer prevention with fertility preservation. However, robust biomarkers for accurate risk stratification remain limited. Although prophylactic HPV vaccination is well established as a primary prevention strategy, its potential therapeutic or protective role following a CIN2 diagnosis remains insufficiently explored.

Therefore, this study aims to identify risk factors associated with disease progression among women with CIN2 undergoing active surveillance, with the goal of informing improved, risk-stratified management strategies. Particular emphasis is placed on evaluating the association between prophylactic HPV vaccination administered after CIN2 diagnosis and patients’ subsequent clinical outcomes.

## Materials and methods

### Study design and population

This was a single-center, retrospective cohort study conducted at Beijing Chaoyang Hospital, Capital Medical University. The study protocol received ethical approval from the Institutional Review Board of Beijing Chaoyang Hospital, Capital Medical University (Approval No.2025-ke-401). We included women who were diagnosed with CIN2 between January 2012 and March 2021 and chose to undergo active surveillance. The inclusion criteria were as follows: (1) age ≥ 18 years, (2) initial CIN2 diagnosis confirmed by at least two independent gynecologic pathologists, and (3) availability of complete clinical and follow-up data for at least 6 months. The exclusion criteria were as follows: (1) pregnancy or lactation at diagnosis; (2) known systemic autoimmune diseases (e.g., systemic lupus erythematosus) or long-term use of immunosuppressive medications for autoimmune conditions; (3) history of any malignancy; (4) prior surgical treatment for cervical HSILs, AIS, or cancer; and (5) incomplete medical records, particularly missing HPV vaccination status or key follow-up visit data.

Additionally, patients with lesions deemed unsuitable for conservative management—specifically, those with high-grade lesions involving more than two quadrants (approximately 50% of the cervical area) on colposcopy or those in whom CIN3+ could not be ruled out despite a CIN2 biopsy—were excluded from this active surveillance cohort.

### Data collection

Baseline demographic and clinical data were extracted from the electronic medical record system. HPV vaccination status was assessed during patient follow-up visits by verifying vaccination information against patient-provided records, a process performed and documented by the attending physician. This verification confirmed whether the patient had received prophylactic HPV vaccination, along with the specific vaccine type, number of doses, and time of administration. The exposure window before and after diagnosis was defined relative to the date of histological CIN2 confirmation. Patients who completed the first vaccine dose before the CIN2 diagnosis date were categorized as “pre-diagnosis” vaccinated, with no minimum interval required. Those who initiated vaccination thereafter were categorized as “post-diagnosis” vaccinated.

### HPV testing and cytology

Cervical specimens were collected using a standardized procedure by physicians and placed into ThinPrep® PreservCyt Solution (Hologic, Inc., Marlborough, MA, USA). Automated liquid-based cytology (LBC) slide processing and preparation were performed using the ThinPrep Pap Test workflow (Hologic). The results were reported according to the Bethesda System terminology.

HPV genotyping was performed using the Liferiver (China) real-time polymerase chain reaction (PCR) system, which is designed to detect high-risk HPV (HR-HPV) genotypes. The assay identifies HPV types 16 and 18 individually, along with additional HR-HPV types, including 31, 33, 35, 39, 45, 51, 52, 56, 58, 59, 66, and 68.

### Colposcopy and histopathology

Colposcopy was performed by experienced colposcopists in accordance with standardized clinical procedures. Targeted biopsies were obtained from the most severe-appearing lesions in the abnormal cervical areas. Specimens collected from different sites were accurately labeled and fixed in 4% neutral buffered formaldehyde. To address cases where the transformation zone (TZ) was not fully visible, or for patients with high-risk factors for endocervical involvement, endocervical curettage (ECC) was performed to reduce the risk of missing occult high-grade lesions. To ensure diagnostic adequacy, only biopsy specimens with a minimum diameter of 3 mm were included, as this size provides sufficient material for routine histopathological evaluation. The histological diagnosis of CIN was established based on morphological features observed in hematoxylin and eosin-stained sections, following the WHO Classification of Tumours of Female Reproductive Organs (4th Edition, 2014). In diagnostically challenging or equivocal cases, p16 immunohistochemistry was used as an ancillary tool to enhance diagnostic accuracy. p16 expression was not analyzed as an independent predictor of disease progression in this study. Pathological diagnoses from different biopsy sites were reported separately, with each site specified individually, particularly when the lesion grade differed. The initial CIN2 diagnosis was confirmed by two expert gynecopathologists who independently and blindly reviewed all histological slides. In cases of disagreement, a joint review was conducted. If consensus could not be reached, a third senior gynecopathologist was consulted to adjudicate, ensuring that the final diagnosis was supported by at least two pathologists.

### Follow-up and outcomes

Patients were allowed to undergo conization and discontinue surveillance at any point. Follow-up evaluations were scheduled every 6 months and included gynecological examination, HR-HPV test, cytology, and colposcopy. Repeat biopsies were performed if (1) cytology results were ≥ ASC-H or indicated glandular cell abnormalities, (2) there was persistent HR-HPV positivity with an abnormal colposcopic impression, or (3) the colposcopic findings indicated progression of cervical disease. After two consecutive negative results for both LBC and HR-HPV testing, patients transitioned to annual follow-up. To minimize loss to follow-up, patients who missed scheduled appointments were actively contacted and encouraged to return for continued monitoring.

### Outcome definitions

Primary Outcome: Complete regression was defined as two consecutive follow-up visits (interval ≥6 months) with negative results for both HR-HPV testing and LBC (defined as negative for intraepithelial lesion or malignancy, NILM).

Secondary Outcomes: Incomplete regression was defined as histologically confirmed CIN1; disease persistence was defined as a persistent histological diagnosis of CIN2 for ≥ 12 months; and disease progression was defined as a histological diagnosis of CIN3, AIS, or invasive CC. In such cases, study withdrawal and timely surgical intervention were recommended.

### Statistical analysis

Data were analyzed using IBM SPSS Statistics Version 26.0. Categorical variables were analyzed using the *χ*^2^ test or Fisher’s exact test and are presented as numbers (percentages). Continuous variables are expressed as mean ± standard deviation. To comprehensively evaluate factors associated with disease progression, two complementary statistical approaches were used. First, to identify independent factors associated with progression, univariate logistic regression analyses were conducted. Variables with a *p*-value of < 0.1 in the univariate analysis were considered candidates for inclusion in the initial multivariate logistic regression model. The final multivariate model was developed using a forward stepwise selection based on the likelihood ratio (LR) test, and results are reported as odds ratios (ORs) with 95% confidence intervals (CIs). Second, to account for variations in follow-up duration and assess time-to-progression, Kaplan–Meier time-to-event analysis curves were generated and compared using the log-rank test. A multivariate Cox proportional hazards regression model was subsequently constructed, incorporating the key independent factors identified through logistic regression. The results are presented as hazard ratios (HRs) with 95% CIs. A two-sided *p*-value of < 0.05 was considered statistically significant for all analyses.

## Results

**Figure 1:**
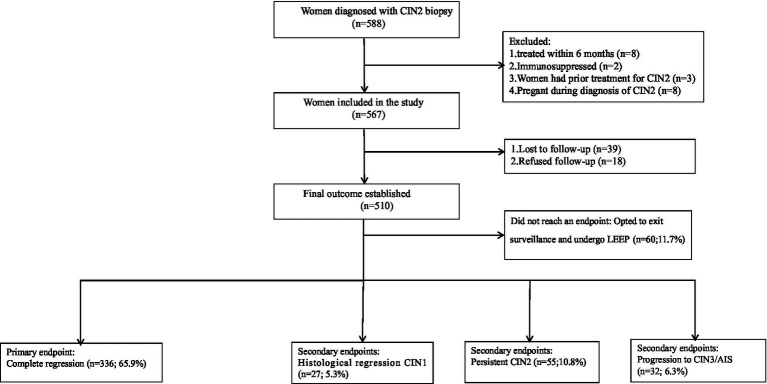
Study flowchart of CIN2 patients managed under active surveillance. This flowchart illustrates the disposition of the 510 patients included in the final analysis (full cohort). Among them, 60 patients opted for LEEP during follow-up prior to reaching a study endpoint and exited surveillance. Clinical outcomes are reported for the remaining 450 patients who continued active surveillance (surveillance cohort). Overall outcome percentages are calculated based on the full cohort (*n* = 510). LEEP, loop electrosurgical excision procedure; CIN2, cervical intraepithelial neoplasia grade 2.

During the study period, a total of 588 women were diagnosed with CIN2. Of these, 78 were excluded for the following reasons: 8 underwent the loop electrosurgical excision procedure (LEEP) within 6 months of the CIN2 diagnosis, 2 had autoimmune diseases (specifically systemic lupus erythematosus, SLE), 3 had received prior surgical treatment for cervical lesions before the current diagnosis, 8 were pregnant at the time of diagnosis, and 57 patients (including 39 lost to follow-up and 18 who declined further participation) had no outcome data available. Consequently, 510 patients were included in the final analysis. All subsequent outcome percentages were calculated based on this full cohort (*n* = 510) according to the intention-to-treat principle, providing an estimate of risk from the point of electing active surveillance. In this cohort, 60 patients (11.7%) chose to discontinue surveillance and underwent LEEP before reaching any study endpoint. Compared with patients who remained in the active surveillance group, those who withdrew for surgery exhibited significant differences in baseline characteristics: they had a higher median age [32.50 years (IQR: 26–38) vs. 29.00 years (IQR: 26–32), *p* < 0.001] and a lower rate of post-diagnosis HPV vaccination [3 of 60 (5.0%) vs. 122 of 450 (27.1%), *p* < 0.001]. No statistically significant differences were observed between the two groups regarding other baseline characteristics, including educational level, smoking status, and HPV genotype, while the remaining 450 patients continued under active surveillance until a definitive endpoint was observed (Figure 1). For the full cohort (*n* = 510), the mean age was 30.15 ± 5.82 years (range: 18–61), and the mean follow-up duration was 37.0 ± 1.3 months. A total of 336 patients (65.9%) achieved complete regression at the final visit, with a median time to regression of 9.1 months (range: 5.1–17.3). Regarding secondary outcomes, histological regression to CIN1 occurred in 27 patients (5.3%), persistent CIN2 was observed in 55 patients (10.8%), and disease progression occurred in 32 patients (6.3%), with a median time to progression of 13.2 months (range: 8.4–21.3). Among the 510 patients, 357 (70.0%) did not receive prophylactic HPV vaccination. Of the 153 vaccinated patients (30.0%), 28 (18.3%) were vaccinated before CIN2 diagnosis, and 125 (81.7%) were vaccinated after diagnosis. The median age at first vaccination was 29.00 years (IQR: 25.0–32.0) for the post-diagnosis group and 27.5 years (IQR: 24.3–31.0) for the pre-diagnosis group (*p* = 0.335). For the pre-diagnosis group, the median interval from vaccination to diagnosis was 9.0 months (IQR: 5.0–24.5). For patients who received vaccination after diagnosis, no detailed vaccination dates were recorded. Based on clinical practice, it was estimated that most of these vaccinations occurred within 6–12 months post-diagnosis. Of the 28 women who received vaccination before diagnosis, none had documented vaccination records prior to age 17. Specifically, 11 patients were vaccinated within 6 months before diagnosis, 10 patients between 6 months and 2 years before diagnosis, and 7 patients more than 2 years before diagnosis. Only one case of disease progression was observed in this subgroup. Due to the limited sample size, only descriptive analyses were performed. All administered vaccines were prophylactic, including 13 who received the 2-valent, 77 received the 4-valent, and 63 received the 9-valent vaccine. Vaccinated patients had a markedly higher overall regression rate of 86.9% (133 of 153) than the unvaccinated patients (56.9%, 203 of 357), with rates by vaccine type as follows: 2-valent: 92.3% (12 of 13), 4-valent: 88.3% (68 of 77), and 9-valent: 84.1% (53 of 63). Pearson’s chi-square test indicated a statistically significant difference in regression rates among the four groups (unvaccinated and three vaccinated subgroups; *χ*^2^(3) = 35.71, *p* < 0.001). However, pairwise comparisons between the different vaccine groups revealed no statistically significant differences: 2-valent vs. 4-valent (*χ*^2^ = 0.18, *p* = 0.672), 2-valent vs. 9-valent (*χ*^2^ = 0.58, *p* = 0.445), and 4-valent vs. 9-valent (*χ*^2^ = 0.52, *p* = 0.472) (Table 1).

**Table 1:** Demographical characteristics and clinical data of the patients.

Variables (category)	Count and percentage (*n* = 510)	95% confidence interval
Age (years)		
Mean ± SD	30.15 ± 5.82	–
Range	18–61	–
Educational level		
Below bachelor’s degree	146/510 (28.6%)	(24.5–32.7)
Bachelor degree or above	364/510 (71.4%)	(67.3–75.5)
Smoking status		
Yes	248/510 (48.6%)	(44.1–52.9)
No	262/510 (51.4%)	(47.0–55.7)
Weekly alcohol use		
Yes	47/510 (9.2%)	(6.9–11.8)
No	463/510 (90.8%)	(88.2–93.1)
Contraception (before diagnosis)		
Condom only	232/510 (45.5%)	(41.2–49.8)
Condom or none	278/510 (54.5%)	(50.2–58.8)
Contraception (after diagnosis)		
Condom only	312/510 (61.2%)	(57.1–65.3)
Condom or none	98/510 (38.8%)	(34.7–42.9)
Age at first intercourse (years)		
(Mean ± SD)	21.24 ± 2.74	–
Number of sexual partners (before diagnosis of CIN2)		
≤1	121/510 (23.7%,)	(20.0–27.5)
>1	389/510 (76.3%)	(72.5–80.0)
Number of sexual partners (after diagnosis of CIN2)		
≤1	383/510 (75.1%)	(71.2–78.6)
>1	127/510 (24.9%)	(21.4–28.8)
Number of pregnancies		
≤3	482/510 (94.5%)	(92.5–96.5)
>3	28/510 (5.5%)	(3.5–7.5)
Symptoms		
Cervical contact bleeding	83/510 (16.3%)	(13.1–19.6)
Others	427/510 (83.7%)	(80.4–86.9)
Menstruation		
Regular menstruation	505/510 (99.0%)	(98.0–99.8)
Menopause	5/510 (1.0%)	(0.2–2.0)
Vaccination		
Yes	153/510 (30.0%)	(25.9–33.9)
No	357/510 (70.0%)	(66.1–74.1)
Vaccination before diagnosis of CIN2		
Yes	28/510 (5.5%)	(3.5–7.5)
No	482/510 (94.5%)	(92.5–96.5)
Vaccination after diagnosis of CIN2		
Yes	125/510 (24.5%)	(20.8–28.3)
No	385/510 (75.5%)	(71.7–79.2)
Previous cytology		
≤ASC-US	360/510 (70.6%,)	(66.6–74.6)
>ASC-US	150/510 (29.4%)	(25.4–33.4)
HPV genotypes		
16+	172/510 (33.7%)	(29.6–37.8)
HR-Others	338/510 (66.3%)	(62.2–70.4)
Number of biopsies		
1	276/510 (54.1%)	(49.8–58.5)
>1	234/510 (45.9%)	(41.5–50.2)

CIN2, cervical intraepithelial neoplasia grade 2; ASC-US, atypical squamous cells of undetermined significance; HPV, human papillomavirus; HR-Others, Other high-risk HPV genotypes, excluding HPV16.

### Risk factors for disease progression

In the univariate analysis, the following factors were significantly associated with disease progression: HPV16 infection (*p* < 0.001) and a history of more than three pregnancies (*p* < 0.001) (Table 2). When all vaccinated patients were analyzed as a single group (irrespective of timing), a protective effect was observed (*p* = 0.034). However, when analyzed separately, vaccination administered after CIN2 diagnosis showed a protective effect that approached statistical significance (*p* = 0.052), whereas vaccination before diagnosis showed no statistically significant effect (*p* = 0.837). Therefore, subsequent multivariate analyses focused on post-diagnosis vaccination (Table 2).

**Table 2:** Univariate and multivariate analysis of factors associated with CIN2 progression.

Variable	Patient distribution, n (%)		Univariate analysis	Multivariate analysis	
	Non-progression (*n* = 478)	Progression (*n* = 32)	*p* value	Adjusted OR (95% CI)	*p* value
Age (years)					
>30	187 (39.1)	15 (46.9)	0.385	–	–
≤30	291 (60.9)	17 (53.1)			
Education					
Below bachelor’s degree	135 (28.2)	11 (34.4)	0.457	–	–
Bachelor degree or above	343 (71.8)	21 (65.6)			
Smoking					
Yes	229 (47.9)	19 (59.4)	0.209	-	-
No	249 (52.1)	13 (40.6)			
Weekly alcohol use					
Yes	42 (8.8)	5 (15.6)	0.195	–	–
No	436 (91.2)	27 (84.4)			
Contraception (before diagnosis)					
Condom only	221 (46.2)	11 (34.4)	0.192	–	–
Condom or non-barrier methods	257 (53.8)	21 (65.6)			
Contraception (after diagnosis)					
Condom only	295 (61.7)	17 (53.1)	0.334	–	–
Condom or non-barrier methods	183 (38.3)	15 (46.9)			
Age at first intercourse (y)					
≥18	451 (94.4)	30 (93.8)	0.887	–	–
<18	27 (5.6)	2 (6.2)			
Number of sexual partners (before diagnosis of CIN2)					
>1	366 (76.6)	23 (71.9)	0.546	–	–
≤1	112 (23.4)	9 (28.1)			
Number of sexual partners (after diagnosis of CIN2)					
>1	118 (24.7)	9 (28.1)	0.663	–	–
≤1	360 (75.3)	23 (71.9)			
Number of pregnancies					
>3	21 (4.4)	7 (21.9)	<0.001	4.38 (1.63–11.79)	0.003^*^
≤3	457 (95.6)	25 (78.1)			
Symptoms					
Cervical contact bleeding	78 (16.3)	5 (15.6)	0.918	–	–
Others	400 (83.7)	27 (84.4)			
Menstruation					
Regular menstruation	474 (99.2)	31 (96.9)	0.730	–	–
Menopause	4 (0.8)	1 (3.1)			
Vaccination status					
Yes	149 (31.2)	4 (12.5)	0.034	–	–
No	329 (68.8)	28 (87.5)			
Vaccination before diagnosis of CIN2					
Yes	27 (5.6)	1 (3.1)	0.837	–	–
No	451 (94.4)	31 (96.9)			
Vaccination after CIN2 diagnosis					
Yes	122 (25.5)	3 (9.4)	0.052	0.29 (0.08–0.98)	0.046^*^
No	356 (74.5)	29 (90.6)			
Previous cytology					
>ASC-US	140 (29.3)	10 (31.2)	0.814	–	–
≤ASC-US	338 (70.7)	27 (68.8)			
HPV genotypes					
16+	152 (31.8)	21 (65.6)	<0.001	4.07 (1.88–8.81)	<0.001^*^
HR others	327 (68.2)	11 (34.4)			
Number of biopsies					
>1	218 (45.6)	16 (50.0)	0.629	–	–
1	260 (54.4)	16 (50.0)			

The final multivariable logistic regression model was constructed using forward stepwise selection (based on the likelihood ratio test). Candidate variables were those with *p* < 0.1 in the univariate analysis. The final model retained only the following three factors: number of pregnancies (>3), HPV vaccination status after CIN2 diagnosis, and HPV16 infection status.; ^*^p < 0.05.

CIN2, Cervical intraepithelial neoplasia grade 2; OR, Odds Ratio; CI, Confidence Interval; ASC-US, Atypical Squamous Cells of Undetermined Significance.

The multivariate logistic regression model confirmed that HPV16 infection (OR 4.07, 95% CI 1.88–8.81, *p* < 0.001) and more than three pregnancies (OR 4.38, 95% CI 1.63–11.79, *p* = 0.003) were independent risk factors for progression. Notably, HPV vaccination after CIN2 diagnosis was associated with a significantly reduced risk of progression (OR 0.29, 95% CI 0.08–0.98, *p* = 0.046), as shown in Table 2. This protective effect was corroborated by the time-to-event analysis. In the multivariate Cox regression analysis, which assessed the risk associated with not being vaccinated, the hazard of disease progression was significantly higher for patients who did not receive the vaccine after diagnosis (HR 3.39, 95% CI 1.02–11.20, *p* = 0.046) compared with those who were vaccinated. In summary, the protective effect of vaccination was supported by both the OR (0.29) and HR (3.39). In Table 3, significant risk factors for progression included women with >3 pregnancies (HR 3.95, 95% CI 1.69–9.26, *p* = 0.002) and those who tested HPV16-positive (HR 3.70, 95% CI 1.77–7.75, *p* < 0.001).

**Table 3:** Progression-free survival analysis by the Cox model.

Factors	Hazards ratio (95% CI)	*p* value
Pregnancy times >3 VS. ≤3	3.95 (1.69–9.26)	0.002
Vaccination after diagnosis of CIN2 Non-recipientsRecipients	3.39 (1.02–11.20)	0.046
HPV genotypes 16+ VS. Other genotypes	3.70 (1.77–7.75)	<0.001

CIN2, Cervical intraepithelial neoplasia grade 2; OR, Odds Ratio; CI, Confidence interval; CI, confidence interval.

### Risk stratification and 5-year progression rates

The overall 5-year disease progression rate (DPR) was 11.8%. For patients with HPV16 infection, more than three pregnancies, and no HPV vaccination after CIN2 diagnosis (*n* = 13, 2.5% of the cohort), the Kaplan–Meier estimated 5-year survival was 72% (5 events of 13 patients). This smallest and highest-risk subgroup warrants cautious interpretation; nevertheless, its markedly high progression rate serves as a critical alert for targeting intensive clinical management. In contrast, among patients with three or fewer pregnancies, who were HPV16-negative and vaccinated after diagnosis (*n* = 497), the 5-year DPR was significantly lower, at 7.6% (27 events of 497 patients).

**Figure 2:**
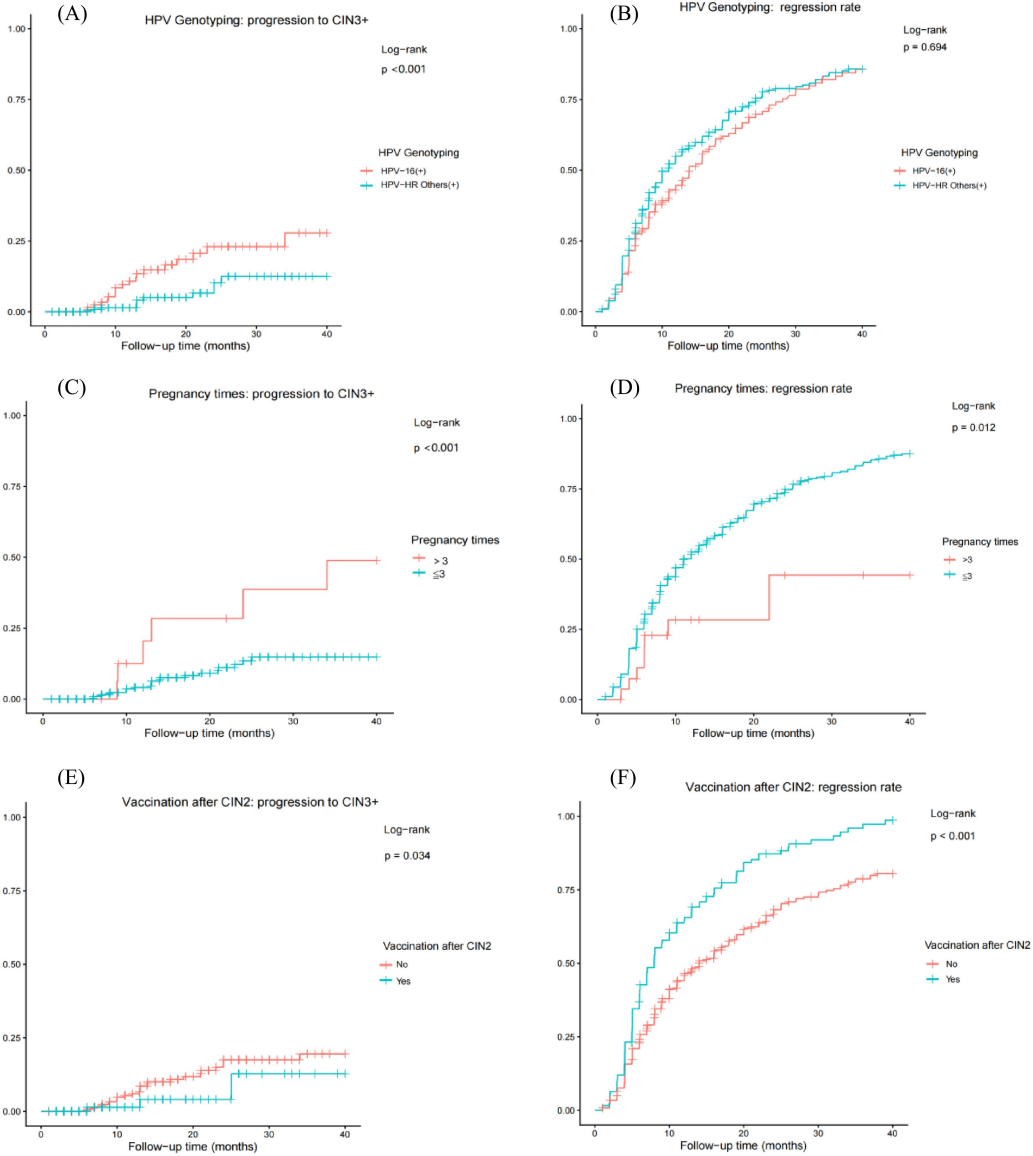
Cumulative incidence curves for disease progression and regression in CIN2 patients stratified by different factors. **(A, C, E)** Cumulative incidence curves for progression to CIN3+, stratified by: **(A)** HPV genotype (16+ vs. other high-risk types); **(C)** Number of pregnancies (>3 vs. ≤3); **(E)** HPV vaccination status after CIN2 diagnosis (yes vs. no). **(B, D, F)** Cumulative incidence curves for complete regression, stratified by the corresponding factors in panels A, C, and E, respectively. Between-group comparisons were made using the log-rank test.

Time-to-Event Analysis for Disease Progression and Regression. The competing outcomes of progression to CIN3+ and spontaneous regression were analyzed using Kaplan–Meier time-to-event analysis over 40 months of follow-up, with between-group comparisons made by the log-rank test (Figure 2).

Patients with baseline HPV16 infection exhibited a significantly higher cumulative incidence of progression (A, log-rank *p* < 0.001). In contrast, although the regression rate appeared higher in patients with other HR-HPV types (non-HPV16), the difference was not statistically significant (B, log-rank *p* = 0.694).

Women with more than three pregnancies were associated with an increased risk of progression (C, log-rank *p* < 0.001) and a decreased likelihood of regression (D, log-rank *p* = 0.012) relative to women with three or fewer pregnancies.

Critically, among women undergoing conservative management for CIN2, receipt of prophylactic HPV vaccination after diagnosis was associated with a significantly reduced cumulative incidence of progression (E, log-rank *p* = 0.034) and a strong trend toward a higher regression rate (F, log-rank *p* < 0.001), compared with the unvaccinated group.

Notably, in clinical practice, some patients returned for follow-up visits earlier than scheduled due to anxiety or other personal reasons, resulting in the premature documentation of outcomes.

## Discussion

To our knowledge, this is the first large-scale, long-term study worldwide investigating the natural history of CIN2 in over 500 patients under active surveillance with a mean follow-up exceeding 37 months, demonstrating a complete regression rate of 65.9% and a progression rate of 6.3%. Our study showed a progression rate notably lower than the 10–18% reported in previous studies (6, 8, 9). This is also the first study to identify post-diagnosis prophylactic HPV vaccination as an independent protective factor against disease progression, through a comparison of viral clearance outcomes among unvaccinated individuals and those vaccinated either before or after CIN2 diagnosis. Moreover, HPV16 infection and having multiple pregnancies were identified as independent risk factors for disease progression.

HPV16 is widely recognized as a critical viral factor contributing to disease persistence and progression. Our findings indicate that HPV16 infection significantly increased the risk of CIN2 progression compared with infections by other genotypes, confirming its status as a critical viral factor, which is consistent with data from contemporary studies (10). This observation aligns with multiple published studies and reflects the disproportionately high prevalence of HPV16 in CIN3 and cervical cancer cases (11). The elevated risk associated with HPV16 is primarily driven by the oncogenic functions of the E6 and E7 oncoproteins (12), which facilitate malignant transformation by degrading tumor suppressor proteins and inducing genomic instability (13, 14). These findings underscore the clinical importance of vigilant monitoring in patients infected with HPV16.

Our data also indicated that more than three pregnancies were associated with an increased risk of CIN2 progression, but the specific intervals between pregnancies were not analyzed. In our cohort, women with more than three pregnancies constituted a relatively small subgroup (5.5%, 28 of 510). Although this warrants cautious interpretation, our findings are consistent with the view that high parity is a risk factor for cervical carcinogenesis (15). According to a local study in Paraguay, the risk of developing CIN2+ was three-fold higher in women with more than four pregnancies than in those with zero or one pregnancy (16). This association between high parity and CIN2+ has been corroborated by additional studies, particularly among younger women. It has been hypothesized that the risk may be further amplified when several full-term pregnancies occur within a relatively short time frame (17). Importantly, the mode of delivery—whether vaginal or cesarean—does not appear to influence the progression of squamous intraepithelial lesions (SILs) (18). During pregnancy, elevated estrogen levels promote eversion of the cervical squamocolumnar junction, resulting in prolonged exposure of the transformation zone to potential carcinogens such as HPV. This mechanism may contribute to the increased risk of disease progression (19). Furthermore, as pregnancy advances, colposcopic assessment becomes increasingly challenging, which may lead to higher rates of diagnostic inaccuracy. Physiological changes—including extensive squamous metaplasia, increased cervical vascularity, and other pregnancy-related morphological alterations—can complicate the distinction between active metaplasia, low-grade CIN, and benign lesions that may present with suspicious features (20).

Our study provides compelling, albeit preliminary, evidence that preventive HPV vaccination may reduce the risk of disease progression among women with established CIN2. Vaccination emerged as an independent protective factor in the multivariate analysis, with unvaccinated individuals demonstrating a significantly higher risk of progression. Although the relatively wide confidence intervals indicate that these results should be interpreted with caution, this study’s findings are based on the largest cohort to date examining outcomes following prophylactic vaccination after a confirmed CIN2 diagnosis. Future validation in larger prospective cohorts remains necessary. Nevertheless, the consistent association observed across multiple statistical models strongly supports the presence of a true protective effect.

Several studies have shown that combining surgical excision with postoperative vaccination significantly reduces the risk of CIN2+ recurrence (21–24). The “SPERANZA” project further proposes that the immunologically activated microenvironment following conization may synergize with vaccination, eliciting a more robust local antibody response and protecting regenerating cervical tissue from reinfection (24). Biologically, this effect may be explained by the high-titer neutralizing antibodies elicited by prophylactic vaccines, which prevent viral attachment to the basement membrane. This process, together with the clearance of antibody–virus complexes by neutrophils (25), reduces the likelihood of reinfection or new infections with the same or phylogenetically related HPV types. Consequently, vaccination may lower the local viral burden and shift the immune environment in favor of lesion regression. Kang et al. identified not receiving post-conization vaccination as an independent predictor of recurrence (21). This hypothesis is supported by previous clinical evidence. Our finding that post-diagnosis vaccination is protective in an active surveillance setting contrasts with studies on surgically treated women, where pre-treatment vaccination appears more effective (26, 27). Sand et al. reported that vaccination before conization reduced the risk of subsequent CIN2+ by 23%, whereas vaccination after conization showed no significant benefit (26). Similarly, Henere et al. found that pre-treatment vaccination significantly reduced post-treatment HSILs, while post-treatment vaccination did not (27). This discrepancy likely reflects different clinical contexts: under active surveillance, the persisting lesion provides ongoing antigenic stimulation, enabling post-diagnosis vaccination to enhance immune-mediated clearance of established HPV infection. In contrast, after surgical excision, the primary benefit of vaccination is the prevention of reinfection, rendering pre-treatment immunization—while the antigen source is still present—theoretically more optimal. These complementary findings suggest that the optimal timing of HPV vaccination may depend on the management strategy, and both approaches warrant consideration in clinical practice.

However, this potential secondary benefit must be clearly differentiated from the mechanism of true therapeutic HPV vaccines, which are currently under development (28). Our initial findings indicate that the protective effect observed even when HPV vaccination was administered following a CIN2 diagnosis suggests a mechanism of action extending beyond conventional primary prevention. This potentially provides novel evidence for fertility preservation. The observed effect of prophylactic vaccination in patients with CIN2, although its precise mechanism remains incompletely understood, aligns with the design principle of licensed prophylactic vaccines, which target the L1 capsid protein and are not designed to clear established infections, as the L1 gene is often disrupted during viral integration. In contrast, therapeutic vaccines aim to induce a cellular immune response against the persistently expressed E6 and E7 oncoproteins, thereby targeting infected and transformed cells directly (29). In the current absence of approved therapeutic vaccines, the potential positive impact of prophylactic vaccination observed in our CIN2 cohort underscores its practical clinical relevance: it offers a feasible and safe interventional option for patients who lack access to therapeutic vaccination. For patients diagnosed with CIN2 who remain unvaccinated, our findings suggest that HPV vaccination may confer dual benefits: preventing new HPV infections and potentially reducing the risk of progression of existing lesions. Therefore, recommending prophylactic HPV vaccination following a CIN2 diagnosis appears to be a reasonable and low-risk adjunct to active surveillance. Nevertheless, prospective randomized controlled trials are needed to validate this effect and refine its role in clinical management.

### Strengths and limitations

Key strengths of our study include a large sample size, an extended follow-up period, standardized diagnostic and surveillance protocols, and comprehensive clinical data collection. To our knowledge, this is the first study specifically designed to investigate the impact of HPV vaccination administered after diagnosis on CIN2 progression.

The limitations include its retrospective study design, which introduces the possibility of selection and information bias. The exact interval from diagnosis to vaccination could not be determined for the post-diagnosis vaccination group. The single-center setting may also limit the generalizability of our study. Additionally, follow-up data from other healthcare institutions could not be obtained for certain patients. Future prospective, multicenter studies are needed to identify more precise predictors of outcomes in this population.

## Conclusion

In conclusion, a risk-stratified management strategy for CIN2 is justified. Active surveillance is a safe and effective approach for the majority of women with CIN2. However, for patients with HPV16 infection and more than three pregnancies, cervical conization should be recommended after completion of childbearing due to their substantially elevated risk of progression. Most importantly, for all unvaccinated individuals diagnosed with CIN2, HPV vaccination administered after diagnosis is strongly recommended, as our study identifies it as an independent protective factor that significantly reduces the risk of progression.

## Data Availability

The original contributions presented in the study are included in the article/supplementary material, further inquiries can be directed to the corresponding author.
